# Time to positivity of blood cultures supports early re-evaluation of empiric broad-spectrum antimicrobial therapy

**DOI:** 10.1371/journal.pone.0208819

**Published:** 2019-01-02

**Authors:** Merel M. C. Lambregts, Alexandra T. Bernards, Martha T. van der Beek, Leo G. Visser, Mark G. de Boer

**Affiliations:** 1 Department of Infectious Diseases, Leiden University Medical Center, Leiden, The Netherlands; 2 Department of Microbiology, Leiden University Medical Center, Leiden, The Netherlands; Medical University of Gdansk, POLAND

## Abstract

**Background:**

Blood cultures are considered the gold standard to distinguish bacteremia from non-bacteremic systemic inflammation. In current clinical practice, bacteraemia is considered unlikely if blood cultures have been negative for 48–72 hours. Modern BC systems have reduced this time-to-positivity (TTP), questioning whether the time frame of 48–72 hrs is still valid. This study investigates the distribution of TTP, the probability of blood culture positivity after 24 hours, and identifies clinical predictors of prolonged TTP.

**Methods:**

Adult patients with monomicrobial bacteremia in an academic hospital were included retrospectively over a three-year period. Clinical data were retrieved from the medical records. Predictors of TTP >24 hours were determined by uni- and multivariate analyses. The residual probability of bacteremia was estimated for the scenario of negative BCs at 24 hours after bedside collection.

**Results:**

The cohort consisted of 801 patients, accounting for 897 episodes of bacteremia. Mean age was 65 years (IQR 54–73), 534 (59.5%) patients were male. Median TTP was 15.7 (IQR 13.5–19.3) hours. TTP was ≤24 hours in 85.3% of episodes. Antibiotic pre-treatment (adjusted OR 1.77; 95%CI 1.14–2.74, p<0.01) was independently associated with prolonged TTP. The probability of bacteremia, if BC had remained negative for 24 hours, was 1.8% (95% CI 1.46–2.14).

**Conclusion:**

With adequate hospital logistics, the probability of positive blood cultures after 24 hours of negative cultures was low. Combined with clinical reassessment, knowledge of this low probability may contribute to prioritization of the differential diagnosis and decisions on antimicrobial therapy. As a potential antibiotic stewardship tool, this strategy warrants further prospective investigation.

## Introduction

Empirical in-hospital antibiotic prescription forms a significant proportion of broad-spectrum antibiotic consumption. The in-hospital use of antibiotics is expected to increase even further due to advancing life-expectancy and an increase in the application of immunosuppressive therapies, resulting in an increasing incidence of bacterial infections [1–3]. Appropriate empirical treatment for severe bacterial infections improves survival [1, 4]. However, upon presentation, the clinical diagnosis is often uncertain, and the presence of bacterial infection is not always evident. There is a broad differential diagnosis for fever, including viral infections and inflammatory states of noninfectious origin such as pancreatitis. Furthermore, thrombo-embolic events and severe drug reactions can mimic the symptoms of bacterial infection [5]. Identifying patients without bacterial infection at an early time point is an important component of antimicrobial stewardship. Prolonged administration of broad-spectrum antibiotics may cause adverse events in the individual patient. Duration of antibiotic therapy is associated with toxicity, *Clostridium difficile* infection and increased mortality rates [6–9]. Furthermore, antibiotic consumption, especially the use of broad-spectrum agents, is one of the major drivers of the increasing antimicrobial resistance worldwide [10, 11]. Because of these individual and societal risks, guidelines recommend to de-escalate broad-spectrum antimicrobial treatment based on the source of infection and culture results. De-escalation of empirical therapy is defined as a reduction in number and/or narrowing of spectrum of antimicrobial agents [12]. When infection is found not to be present, the recommendation is to discontinue antimicrobial therapy [1].

Diagnostics directed at the possible source of infection, i.e. radiographic exams and urine analysis, can be completed within hours. In contrast, differentiating bacteremia from non-bacteremic infection is still time consuming as reliable alternatives for conventional blood culture incubation are not yet available in clinical practice. Biomarkers for exclusion of bacteremia lack sensitivity or have practical limitations [13–16]. Historically, the consensus is to await blood culture results for at least 48 to 72 hours, before bacteremia is deemed unlikely [17, 18]. Because of the modernisation of blood culture methods, and especially the development of continuous monitoring systems, the time to positivity (TTP) of blood cultures has been reduced substantially, see Box 1 [19–21].

Knowledge of the distribution of blood culture TTP is of clinical benefit in the re-evaluation of patients with a clinical syndrome consistent with infection. A low probability of bacteremia when blood cultures have remained negative after 24 hours, may have impact on the differential diagnosis and subsequent diagnostic and therapeutic actions. Our aim was to determine the distribution of the TTP of blood cultures in adult patients and asses the probability of bacteremia when blood cultures have remained negative for 24 hours. In addition, we aimed to identify clinical characteristics that predict late (i.e. >24 hours) positivity.

## Methods

### Setting and study participants

The retrospective cohort study was performed at the Leiden University Medical Center, a tertiary care and teaching hospital in the Netherlands.

All patients aged 18 years and older, with mono-microbial bacteremia in 2013 and 2014 were identified. An additional 100 patients that presented in the year 2015 were randomly included, by case identification code. Patients with polymicrobial bacteremia were excluded as the time to positivity of the individual pathogens was unknown. Furthermore the relevance of the individual pathogens to the TTP of the polymicrobial culture can not be determined.

The blood culture database of the Department of Medical Microbiology was used to identify eligible patients. Patients admitted to clinical wards, including medium and intensive care unit and cases presenting at the emergency department were eligible for inclusion. Multiple episodes of bacteremia per patient were allowed if the antimicrobial therapy for the previous episode had been completed and clinical and microbiological cure had been achieved. All blood cultures with coagulase-negative staphylococci (CoNs) were excluded, because the likelihood that these cultures represent contamination is high. For other possible contaminants (including anaerobes) the differentiation between true bacteremia and contamination was based on the number of positive vials and the documented assessment of the microbiologist and responsible physician.

Standard empiric therapy for sepsis of unknown origin in the study centre was a second generation cephalosporin combined with an aminoglycoside.

The study was approved by the Institutional Ethics Review Board of the Leiden University Medical Center. The requirement to obtain informed consent was waived because of the retrospective nature of the study.

### Data collection

Demographic data, data about pre-existing medical conditions, clinical parameters at presentation, the most likely source of bacteremia and the outcome data were retrieved from the electronic medical records. The classification of the source of infection was based on review of the available clinical, radiological and microbiological information. Outcome measurements included admission to the intensive care, length of hospitalisation and 30-day mortality. Microbiological data, including pathogen identification and TTP, were retrieved from the database of the Department of Medical Microbiology.

Antibiotic pre-treatment was defined as treatment with one or more antibiotic agents, administered intravenously, intramuscularly or orally, within the 24 hours preceding collection of the first blood culture. Oral antibiotics without systemic absorption, such as vancomycin, were excluded from this definition.

### Blood culture handling procedures and laboratory techniques

TTP was defined as the time between collection of the blood cultures and the positive signal in the BACTEC FX continuous monitoring system (Becton Dickinson B.V., Breda). The institutional protocol is to collect both an aerobic and anaerobic vial, and to collect 8–10 ml of blood per vial [22, 23]. A quality assessment in 100 individual vials showed a median blood volume of 9 ml (IQR 7–11) per vial (S2 Table). The time of bedside blood culture collection was recorded in the electronic medical records, as part of the ordering procedure. Cultures were transported to the in-hospital microbiology department by dedicated hospital transportation employees. During day-hours, transportation is performed every 3 hours. Quality assessment at the beginning of the study period showed a median time from collection to placement in the incubator of 94 minutes (IQR 63–137). Outside working hours the maximum time to transportation is 5 hours.

Upon arrival at the Department of Medical Microbiology the blood cultures were directly placed in the BACTEC FX continuous monitoring system (Becton Dickinson B.V., Breda), for a minimum of seven days. The time of the positive signal was automatically recorded.

During evening and night hours, blood cultures were directly placed in the BACTEC, but registration in the system was performed the following morning between 8 and 9 a.m. If the threshold for positivity was reached between placement and this registration, the culture was recorded positive at the time of registration, instead of upon positive signalling. This technical limitation leads to an overestimation of the TTP in ‘unregistered’ bottles. Therefore, median TTP was additionally calculated excluding ‘unregistered’ episodes (S1 Table).

If multiple separate blood cultures from one patient were collected within a time frame of two hours, the shortest TTP was used for the statistical analyses.

### Blood culture positivity rate

To calculate the probability of positive blood cultures when they have remained negative for 24 hours, information on the institutional blood culture positivity rate is required (see statistical analysis). To estimate the overall blood culture positivity rate, the proportion of bacteremia was determined during two separate months, June and December 2014. During this period, all patients in whom blood cultures were obtained because of fever or (suspected) sepsis were included. True bacteremia was defined as growth of a pathogenic bacterial species in ≥1 blood culture bottle. Definition of contamination was identical to the definition applied in the main cohort. Patients were only included for the first episode of suspected infection, subsequent episodes were excluded.

### Statistical analyses

Median TTP and interquartile ranges (IQR) were determined for the complete cohort and for the most frequently isolated pathogens in patients with bacteremia. Median TTP was additionally calculated excluding ‘unregistered’ episodes, because of the potential overestimation of TTP (S1 Table). Normally and non- normally distributed continuous variables were compared by Student’s t test and Mann-Whitney U test, respectively. Univariate risk factor analysis was performed for short (<16 hours) and prolonged TTP (>24 hours), using Chi-square statistical tests. Results were reported as risk ratios (RR) with 95% confidence intervals (95%CI). A multivariate analysis for prolonged TTP was performed and results were reported as adjusted odds ratios (OR with 95%CI). Determinants for the multivariate analysis were selected based on p <0.25 in the univariate analysis.

We applied a generalized estimating equation model to assess the potential effect of repeated measurements by inclusion of multiple episodes of bacteremia for a proportion of patients.

The residual risk of detection of bacteremia after 24 hours was calculated applying a previously published mathematical equation [20] (S1 Box). This equation is based on the proportion of positive blood cultures in suspected sepsis and the proportion of blood cultures with prolonged TTP.

All statistical analyses were performed with the IBM SPSS Statistics, version 23.

## Results

### Study population characteristics

After exclusion of polymicrobial and contaminated blood cultures, a total of 801 individual adult patients was included, representing 897 episodes of bacteremia. Mean age was 65 years (IQR 54–73), 534 (59.5%) patients were male.

The majority of bacteremia episodes (511 episodes, 57.0%) was caused by a Gram-negative pathogen, predominantly *Escherichia coli* (263/511, 51.5%). *Streptococcus spp* were the most common Gram-positive isolates (163/386, 42%). The demographic and clinical characteristics of the 897 episodes of bacteremia are summarized in Table 1.

**Table 1:** Demographic and clinical characteristics among 897 episodes with bacteremia.

Characteristic	n = 897 (100%)
**Patient demographics**	
Male gender	534 (59.5)
Age (years), (median, IQR)	65 (54–73)
**Medical history**	
Diabetes mellitus	188 (21.0)
Corticosteroid therapy (prior 6 months)	276 (30.8)
Neutropenia	113 (12.6)
Solid organ transplantation	116 (12.9)
Solid malignancy	170 (19.0)
haematological malignancy	96 (10.7)
Dialysis (haemodialysis/peritoneal dialysis)	20 (2.2)
**Clinical presentation**	
Fever (temperature>38.5 °C)	538 (60.0)
Systolic blood pressure (mmHg) (median, IQR)	125 (107–142)
Pulse rate (bpm) (median, IQR)	101 (88–115)
EMV <15	173 (19.3)
PITT Bacteremia score (median, IQR)	1 (0–2)
Quick SOFA-score (median, IQR)	1 (1–2)
**Antibiotic pre-treatment**	264 (29.4)
**Location of presentation**	
Emergency department	507 (56,5)
General ward	340 (37,9)
ICU/MCU	50 (5.6)
Hospitalization before BC (hours) (median IQR)	3.0 (0.4–136.8)
**Microbiological parameters**	
Gram-positive bacteremia:	386 (43.0)
Gram-negative bacteremia	511 (57.0)
Anaerobic bacteremia	37 (4.1)
**Source of infection**	
Gastro-intestinal	245 (27.3)
Respiratory	89 (9.9)
Endovascular (e.g. thrombus)	111 (12.4)
Urinary tract	232 (25.9)
Skin and soft tissue	71 (7.9)
Other	56 (6.2)
Not identified	84 (9.4)
**Outcome**	
ICU/MCU admission during hospitalization	180 (20.1)
Hospitalization after BC (days) (median IQR)	8.9 (3.9–19.0)
30-day mortality	134 (14.9)

BC = blood culture, ICU/MCU = intensive care unit / medium care unit, IQR = interquartile range

In 450/897 (50.2%) episodes ≥2 blood culture sets were obtained within a time frame of two hours.

### Time to positivity

**Fig 1:**
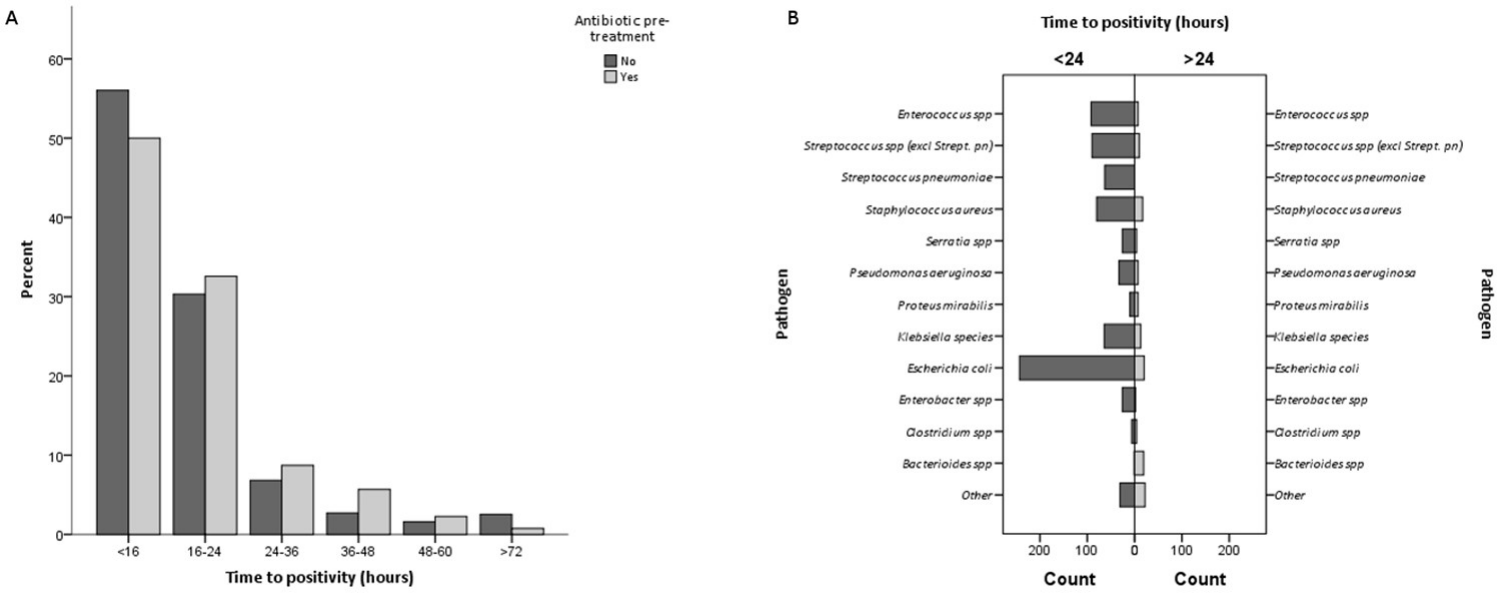
Distribution of time to blood culture positivity (TTP) in 897 episodes of bacteremia. Fig. 1A illustrates the distribution of TTP in patients with and without antibiotic pre-treatment at the time blood cultures were collected. Fig. 1B. illustrates the distribution of TTP, short (≤24) versus prolonged (>24) TTP, according to isolated pathogen. The group ‘Other’ comprises *Citrobacter spp*. *Haemophilus spp*, *Listeria spp*, *Achromobacter spp*, *Acinetobacter spp*, *Moraxella catarrhalis*, *Morganella morganii*, *Propionibacterium acnes*, *Rothia mucilaginosa*, *Salmonella spp*, *Stenotrophomonas maltophilia*, *Lactobacillus spp*, *Prevotella spp*, *Fusobacterium spp*.

The median TTP was 15.7 hours (IQR 13.5–19.3). The TTP was below 24 hours in 765 episodes (85.3%). In 34 (3.8%) episodes and 18 (2.0%) episodes TTP was longer than 48 hours and 72 hours, respectively (Fig 1).

Anaerobic bacteremia was frequent in the prolonged TTP group, 28/132 (21.2%) episodes. After exclusion of anaerobic bacteraemia, there was no statistically significant difference in TTP between Gram-negative and Gram-positive bacteremia (TTP 18.6 h vs 19.4 h, p = 0.48).

**Fig 2:**
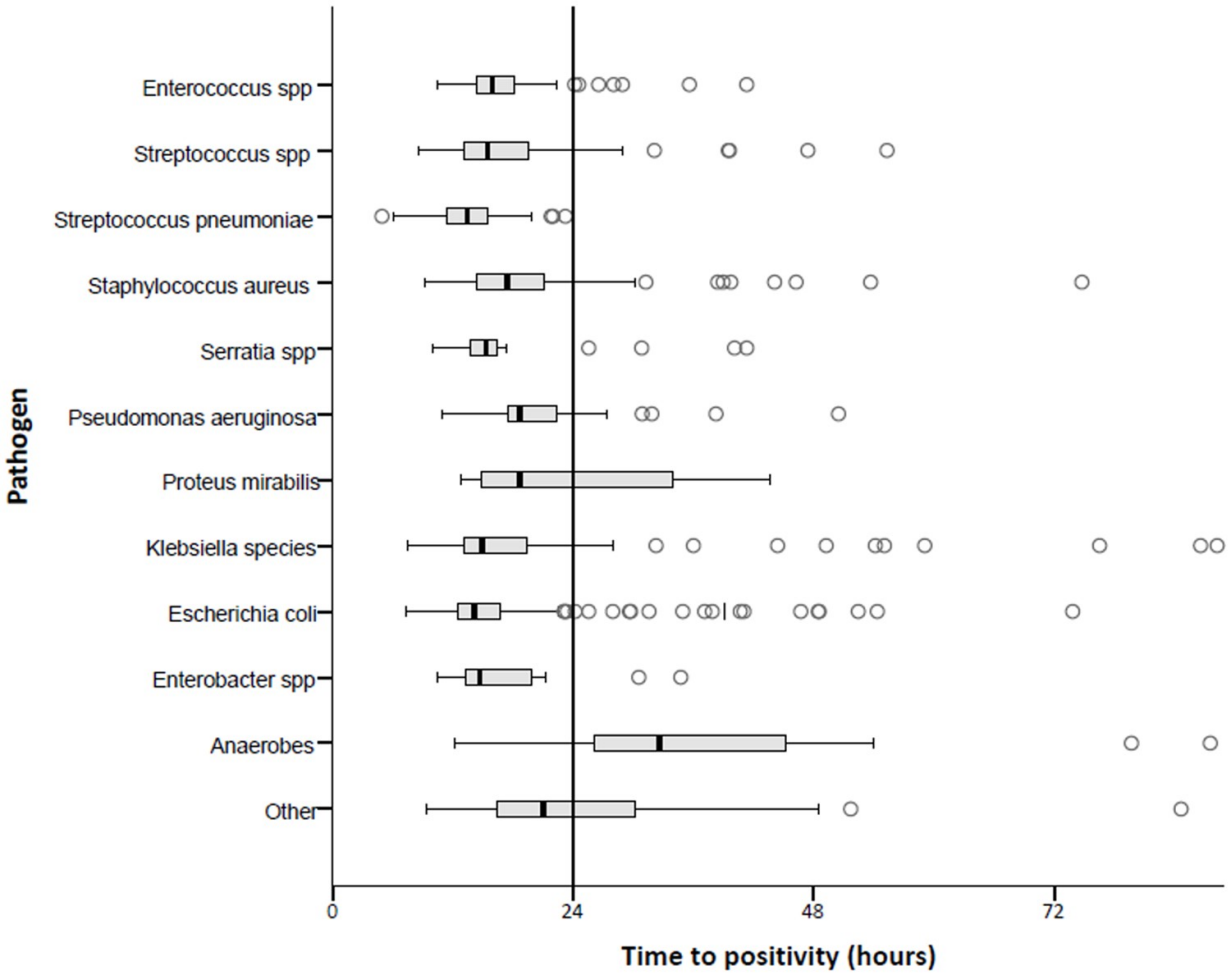
Pathogens and time to positivity (TTP) distributions. The boxplot figure illustrates the distribution of TTP (median, interquartile range) for the most frequently isolated pathogens. The ends of the whiskers represent one and a half times the http://www.statisticshowto.com/probability-and-statistics/interquartile-range/ interquartile range. The group ‘Other’ comprises *Citrobacter spp*. *Haemophilus spp*, *Listeria spp*, *Achromobacter spp*, *Acinetobacter spp*, *Moraxella catarrhalis*, *Morganella morganii*, *Propionibacterium acnes*, *Rothia mucilaginosa*, *Salmonella spp*, *Stenotrophomonas maltophilia*, *Lactobacillus spp*, *Vibrio spp*.

The TTP of the most common pathogens is illustrated in Fig 2. All episodes of *Streptococcus pneumoniae* bacteremia were diagnosed within 24 hours (median 13.4 h, IQR 11.3–15.5 h). TTP was long in bacteremia caused by *Proteus mirabilis* (median 18.6 hr, IQR 14.8–34.9 h). All cases (n = 3) of *Propionibacterium acnes* bacteremia were diagnosed after 72 hours.

In 87 of the 132 (65.9%) episodes with prolonged TTP, the isolated pathogen was susceptible to the institutions empirical sepsis therapy (2nd generation cephalosporin and an aminoglycoside).

In 108 (12.0%) episodes blood cultures were placed in the incubator ‘unregistered’ and reached the threshold for positivity before registration. TTP analysis excluding these episodes did not have an important effect on the results (S1 Table).

### Predictors of short versus prolonged time to positivity

Neutropenia (RR 0.22, 95%CI 0.08–0.58, p <0.01) and corticosteroid therapy (RR 0.66, 95%CI 0.45–0.97, p = 0.03) were associated with short TTP (≤24 hours) in univariate analysis (Table 2). The source of infection was not a predictor of short versus prolonged TTP. In multivariate analysis, antibiotic pre-treatment (adjusted OR 1.71 95%CI 1.11–2.65, p<0.01) was associated with prolonged TTP (> 24 hours). Neutropenia (adjusted OR 0.15 95%CI 0.05–0.43, p<0.01), was associated with short TTP. Application of a generalized estimating equations model did not detect a relevant effect of including >1 episode in a proportion of patients.

**Table 2:** Univariate and multivariate analysis for long time-to-positivity (>24 hours) in 897 episodes of bacteremia.

characteristic	Univariate analysis			Multivariate analysis		
	RR	95% CI	p-value	OR	95% CI	p-value
**Patient demographics**						
Male gender	0.98	0.71–1.35	0.91			
Age > 70 years	1.22	0.88–1.69	0.23	1.15	0.76–1.72	0.52
**Medical history**						
Immunocompetent	1.00	-	-			
Neutropenia	0.21	0.08–0.56	<0.01	0.15	0.05–0.43	<0.01
Corticosteroid therapy	0.64	0.44–0.94	0.02	0.71	0.45–1.14	0.16
**Clinical presentation**						
Temperature>38.5 °C	0.80	0.58–1.11	0.18	0.79	0.54–1.17	0.24
Systolic blood pressure <100 mmHg	1.29	0.86–1.94	0.23	1.09	0.65–1.80	0.73
PITT bacteremia score≥2	0.85	0.58–1.25	0.41			
Quick SOFA score	1.10	0.69–1.75	0.78			
Antibiotic pre-treatment	1.23	0.92–1.77	0.15	1.71	1.11–2.65	0.01
Emergency department	0.77	0.56–1.05	0.10	0.71	0.47–1.07	0.10
**Source of infection**						
Gastro-intestinal	1.29	0.92–1.79	0.14	1.45	0.95–2.22	0.08
Respiratory tract	0.66	0.35–1.26	0.17	0.79	0.38–1.66	0.53
Endovascular	0.78	0.45–1.32	0.34			
Urinary tract	0.99	0.69–1.43	0.97			
Skin and soft tissue	0.95	0.52–1.73	0.88			

CI = confidence interval. RR = relative risk. OR = adjusted odds ratio. CI = confidence interval. The PITT bacteremia score is calculated from temperature of 35.1–36.0°C or 39.0–39.9°C (1 point), temperature of ≤35°C or ≥40°C (2 points), mental status (alert, 0 points; disoriented, 1 point; stuporous, 2 points; comatose, 4 points), hypotension (2 points), receipt of mechanical ventilation (2 points) and cardiac arrest (4 points). The Quick SOFA score is calculated from glascow coma scale <15 (1 point), Respiratory rate ≥22 (1 point), systolic blood pressure ≤100 (1 point).

#### Probability of bacteremia at T = 24 hours

The two determinants in the equation of the probability of bacteremia at T = 24 hours are the blood culture positivity rate and the proportion of blood cultures that is positive within 24 hours (S1 Box). The rate of blood culture positivity was determined during two separate months, June and December 2014. In this period 2,099 blood cultures in 778 patients were obtained because of suspected bacterial infection. In 83/778 episodes one or multiple blood cultures were positive, resulting in a positivity rate of 10.7%.

The probability of bacteremia after 24 hours was calculated using the above estimated overall a priori risk of bacteremia in patients with suspected infection (10.7%), and the fraction of blood cultures that were positive within 24 hours (85.3%) [20]. The probability of bacteremia when blood cultures had remained negative after 24 hours was 1.8% (95% CI 1.46–2.14%).

## Discussion

We found that under the condition of adequate hospital logistics and by using modern, continuously monitoring blood culture systems, 85.3% of blood cultures is positive within 24 hours. Neutropenia was a predictor of short TTP in our study and antibiotic pre-treatment was a predictor of prolonged TTP. These predictors are in line with results from a study by Martinez et al [21]. Most previous studies have defined TTP as the time between incubation and positivity. To permit clinical applicability of the results, we here defined TTP as time between collection of the blood samples and blood culture positivity, taking into account the transportation and laboratory logistics during and outside office hours. As a result, median TTP in our study is longer than in most previous studies [21, 24], but applicable to real-life clinical settings.

For daily practice, the proportion of blood cultures that becomes positive after different periods of elapsed time is more relevant than median TTP. Two previous studies, that included smaller numbers of patients, found similar results on TTP distribution, despite the above mentioned differences in definition [18, 25]. The authors of these studies conclude that their findings support antibiotic de-escalation after 48 hours. However, to decide on the optimal timing of re-evaluation of the differential diagnosis, the more relevant question is how probable bacteremia still is when blood cultures have remained negative at different time points. For that purpose, knowledge about the overall blood culture positivity rate, i.e. the pre-test probability, is essential. The blood culture positivity rate in our centre is 10.7%. This is in line with literature on the prevalence of bacteremia in localised bacterial infection and systemic inflammatory response syndrome (SIRS) [26–28]. By using the previously published mathematical equation (S1 Box), the probability of blood culture positivity after 24 hours is below 2 percent in our institution [20].

This probability is centre specific, as both variables in the mathematical equation may vary between institutions. The first variable, the overall blood culture positivity rate, is dependent on the patient population and the criteria that are applied by doctors to order blood cultures. For example, a ‘culture of culturing’ will result in low blood culture positivity rates. The second variable, the proportion of blood cultures that is positive within 24 hours, is dependent on hospital logistics. If there is an important delay in transportation of the cultures to the laboratory or placement in the incubator, TTP according to our definition, will be longer. The mathematical equation, allows for the calculation of an institution specific probability of blood culture positivity at T = 24 hours.

With adequate hospital logistics, the overall probability of positive blood cultures at T = 24 hours is low. This knowledge is valuable for the differential diagnosis and management of patients with suspected bacterial infection. For example, in the scenario of a confirmed source of infection (e.g. pneumonia), and clinical recovery, preliminary negative blood culture results may support an early intravenous-oral switch [29]. Alternatively, when there are no signs of localised infection and blood cultures are still negative after 24 hours, bacteremia becomes unlikely. This knowledge should prompt timely diagnostic steps into non-bacterial causes of fever that require interventions, as e.g. Influenza, thrombo-embolic events or drug-reactions.

Despite low probabilities a blood culture may incidentally become positive after more than 24 hours. Furthermore, negative blood cultures do not exclude bacteraemia. Nor does the absence of positive blood cultures exclude non-bacteremic infections. Despite this level of uncertainty, re-evaluation of empirical therapy is in place when the probability of bacterial bloodstream infection changes. Re-evaluation of clinical stability, response to empirical therapy and an update of the differential diagnosis, is essential when balancing the potential costs and benefits of de-escalating empiric therapy.

For the application of the findings to clinical practice, it is also important to emphasize that the pre-test probability of bacteremia is variable, not only between institutions, but between patients as well [28]. For example, in the severely ill patient with septic shock, the blood culture positivity rate is higher, and TTP may be shorter, both affecting the bacteremia probability after 24 hours [30]. In the severely ill patient without an alternative diagnosis, even a low probability of bacteraemia or non-bacteremic bacterial infection may warrant continuation or even escalation of broad-spectrum antibiotic therapy.

To the best of our knowledge, we present the largest cohort of patients investigating the distribution of TTP. More importantly, this is the first study to approach TTP of blood cultures from a clinical perspective, providing insight into the probability of bacteremia at the 24 hour time point. A limitation of the present study is that patients with polymicrobial bacteremia where excluded. Based on previous research [31, 32] and on theoretical grounds, the TTP of polymicrobial episodes is comparable to monomicrobial bacteremia, possibly even shorter. Therefore, inclusion of these episodes would at most reduce the probability of blood culture positivity at T>24 hours.

Secondly, the volume of blood collected in the vials was not recorded in the individual cases, and in a proportion of patients only 1 vial-set was collected. In daily practice TTP and the yield of blood cultures could be improved by further optimising specimen collection; specifically vial filling and number of vials [22, 23, 33]. Thirdly, we were not informed about the individual vial transportation times to the microbiology laboratory. However, our institutional transport logistics and transport times are in line with current guidelines and comparable to other institutions [34, 35]. Previous research has shown that transport and incubation of blood cultures outside laboratory reduces turnaround time and accelerates therapeutic interventions [36]. As blood culture collection and transportation procedures impact TTP, audit of blood culture logistics is probably a prerequisite for translation of our results to other institutions.

If modern blood culture systems are used in combination with adequate logistics, the probability of positivity when blood cultures are negative after 24 hours is very low. Postponing re-evaluation of the differential diagnosis, solely for the reason of pending blood culture results, is not rational at this time point. The search for alternative causes of fever can be initiated more rapidly if the probability of bacteremia is incorporated in clinical reasoning. This may lead to better timed de-escalation, iv to oral switch and earlier hospital discharge. The safety as well as the benefits of this antibiotic stewardship opportunity should be subject of future clinical trials.

## Supporting information

S1 Box Formula for the Estimation of probability of bacteremia after 24 hours. (DOCX)

S1 Table Pathogen distribution and median time to positivity excluding and including ‘unregistered’ episodes. (DOCX)

S2 Table Results of the quality assessment of blood volume in blood culture vials. (DOCX)
