# Supplementary material for: Time to positivity of blood cultures supports early re-evaluation of empiric broad-spectrum antimicrobial therapy
Source: PLoS One. 2019 Jan 2;14(1):e0208819. doi: 10.1371/journal.pone.0208819 (PMC6314566; doi:10.1371/journal.pone.0208819)
Supplement: S1 Box — (DOCX) [file pone.0208819.s001.docx]

**S1 Box. Formula for the Estimation of probability of bacteremia after 24 hours.**

P =$\frac{(1-\mathrm{TTP}e ) * X}{1 -\left( \mathrm{TTP}e * X \right)} *$ 100%

P = Probability of a positive blood culture when the sets have remained negative 24 hours after bedside collection.

X = The proportion of patients with positive blood cultures among all patients in whom blood cultures are obtained for suspected bacterial infection (centre-specific).

TTP_e_ = proportion of positive blood cultures that are positive in ≤24 hours.

***Example***

*In the Leiden University Medical Center an estimated 10.6% of patients in whom blood cultures were obtained have true bacteremia and 85.3% (95%CI 83.0-87.6) of blood cultures is positive within 24 hours.*

*Then (use formula, X = 0.11 TTP_e_ = 0.85) If BCs are still negative after 24 hours of incubation, the probability that the culture will become positive is approximately 1.8% (95%CI 1.5-2.1).*

This formula is reproduced from an earlier publication in the European Journal of Haematology. [1]

**Reference**

1. Lambregts MMC, Warreman EB, Bernards AT, Veelken H, von dem Borne PA, Dekkers OM, et al. Distribution and clinical determinants of time-to-positivity of blood cultures in patients with neutropenia. Eur J Haematol. 2017.
