# Supplementary material for: Time to positivity of blood cultures supports early re-evaluation of empiric broad-spectrum antimicrobial therapy
Source: PLoS One. 2019 Jan 2;14(1):e0208819. doi: 10.1371/journal.pone.0208819 (PMC6314566; doi:10.1371/journal.pone.0208819)
Supplement: S1 Table — (DOCX) [file pone.0208819.s002.docx]

**S1 Table. Pathogen distribution and median time to positivity excluding and including ‘unregistered’ episodes.**

| **Pathogen** | **Number of episodes (%)** | **Median TTP (hours) (IQR)** |
| --- | --- | --- |
| Overall | 790 (100.0) | 16.1 (13.3-20.0) |
| Gram-positive pathogen^a^: | 346 (43.8) | 16.3 (13.6-19.8) |
| *Streptococcus spp* | 152 (19.2) | 14.5 (12.4-18.5) |
| *Strept. pneumoniae* | 52 (6.6) | 13.4 (11.4-15.2) |
| *Enterococcus spp.* | 88 (11.1) | 16.5 (14.5-18.5) |
| *Staphylococcus aureus* | 91 (11.5) | 17.7 (14.4-21.7) |
| *Other* | 15 (1.9) | 26.4 (21.3-94.8) |
| Gram-negative pathogen^a^: | 417 (52.8) | 15.5 (13.1-19.3) |
| *Escherichia coli* | 218 (27.6) | 14.1 (12.5-17.5) |
| *Pseudomonas aeruginosa* | 38 (4.8) | 19.2 (17.6-22.4) |
| *Enterobacter spp.* | 26 (3.3) | 15.0 (12.8-20.5) |
| *Klebsiella spp.* | 66 (8.4) | 15.5 (13.2-20.3) |
| *Serratia marcesens* | 25 (3.2) | 15.7 (13.8-16.9) |
| *Proteus mirabilis* | 15 (1.9) | 15.8 (14.7-35.9) |
| Other | 29 (3.7) | 18.2 (15.9-24.2) |
| Anaerobic pathogen | 27 (3.4) | 32.6 (24.1-46.6) |

In 108 (12.0%) episodes blood cultures were placed in the incubator ‘unregistered’ and reached the threshold for positivity before registration. TTP analysis excluding these episodes did not have an important effect on the results compared to the analysis that included all episodes.

**Table A. Pathogen distribution and median time to positivity excluding unregistered episodes.**

**Legend**: TTP= Time to positivity, IQR= inter quartile range. ^a^: aerobes only.

|  |  |  |
| --- | --- | --- |

**Table B. Pathogen distribution and median time to positivity Including unregistered episodes.**

| **Pathogen** | **Number of episodes (%)** | **Median TTP (hours) (IQR)** | **Maximum TTP (hours)** |
| --- | --- | --- | --- |
| Overall | 897 (100) | 15.3 (13.3-19.5) | 303.6 |
| Gram-positive pathogen^a^: | 374 (41.7) | 15.7 (13.5-19.3) | 303.6 |
| *Streptococcus spp* | 163 (18.2) | 14.5 (12.5-18.3) | 55.3 |
| *Strept. pneumoniae* | 63 (7.0) | 13.4 (11.3-15.5) | 23.2 |
| *Enterococcus spp.* | 99 (11.0) | 16.0 (14.3-18.2) | 41.3 |
| *Staphylococcus aureus* | 96 (10.1) | 17.4 (14.4-21.4) | 74.8 |
| *Other* | 16 (1.8) | 26.2 (21.1-92.2) | 303.6 |
| Gram-negative pathogen^a^: | 486 (54.2) | 15.0 (13.0-18.5) | 162.3 |
| *Escherichia coli* | 263 (29.3) | 14.1 (12.6-16.7) | 162.3 |
| *Pseudomonas aeruginosa* | 40 (4.6) | 18.6 (17.5-22.3) | 50.5 |
| *Enterobacter spp.* | 28 (32.2) | 14.7 (13.1-20.2) | 34.7 |
| *Klebsiella spp.* | 77 (8.6) | 15.0 (13.1-19.6) | 116.1 |
| *Serratia marcesens* | 30 (3.3) | 15.3 (13.8-16.5) | 41.3 |
| *Proteus mirabilis* | 17 (1.9) | 18.6 (14.8-34.9) | 43.6 |
| Other | 31 (3.5) | 18.1 (15.5-24.1) | 48.6 |
| Anaerobic pathogen | 37 (4.1) | 32.6 (24.1-46.6) | 129.4 |

**Legend**: TTP= Time to positivity, IQR= inter quartile range. ^a^: Aerobes only.
