# Supplementary material for: Time to positivity of blood cultures supports early re-evaluation of empiric broad-spectrum antimicrobial therapy
Source: PLoS One. 2019 Jan 2;14(1):e0208819. doi: 10.1371/journal.pone.0208819 (PMC6314566; doi:10.1371/journal.pone.0208819)
Supplement: S2 Table — (DOCX) [file pone.0208819.s003.docx]

**S2 Table. Results of the quality assessment of blood volume in blood culture vials.**

Vial volume was measured in 50 anaerobic vials and 50 aerobic vials. Vial volume was below 8 ml in 32/100 (32.0%) of vials and below 7 ml in 15/100 vials (15.0%).

**Table 1. Results of the quality assessment of blood volume in blood culture vials.**

| **Blood volume/vial**  **in millilitre** | | **Frequency**  **(%)** | **Cumulative**  **Percentage** |
| --- | --- | --- | --- |
|  | 4 | 3 (3.0) | 3.0 |
|  | 5 | 7 (7.0) | 10.0 |
|  | 6 | 5 (5.0) | 15.0 |
|  | 7 | 17 (17.0) | 32.0 |
|  | 8 | 15 (15.0) | 47.0 |
|  | 9 | 13 (13.0) | 60.0 |
|  | 10 | 5 (5.0) | 65.0 |
|  | 11 | 13 (13.0) | 78.0 |
|  | 12 | 5 (5.0) | 83.0 |
|  | 13 | 4 (4.0) | 87.0 |
|  | 14 | 4 (4.0) | 91.0 |
|  | 15 | 4 (4.0) | 95.0 |
|  | 16 | 3 (3.0) | 98.0 |
|  | 17 | 1 (1.0) | 99.0 |
|  | 18 | 1 (1.0) | 100.0 |

**Legend**: Volume of blood in 100 BACTEC blood culture vials (Becton Dickinson B.V., Breda).
